# My Failures

**Published:** 1884-08

**Authors:** W. D. Danlap


### My Failures.

BY W. D. DUNLAP.

Read before the Alabama Dental Society.

This truly is an age of rapid and wonderful progress—all the resources of art and science are laid under tribute to hasten the time when we may exclaim "Eureka!"—and it really seems that truth in all its beauty must perforce surrender to the combined powers of investigation. But ever and anon it may be well to take a retrospect, and see if all we claim is based on the solid foundation of truth; otherwise it may be that, after weary hours of toil and trouble, and days of study cheered by bright visions of success, we may have to retrace our steps, and climb again the rugged path to knowledge. There is every day and hour a demand for something new, and the demand must be supplied, let the cost be what it may; old things and methods are ignored, and but little can be found that is not gilded and burnished until it shines as the noonday sun, all novelty and glitter. It would ill become us to deny the good that have these qualities added to real merit, except in cases where good taste is violated for the sake of show. The efforts to produce something new have imperceptibly led us to ignore much that is good in the old. In fact, we have a new Code—a ponderous volume—an encyclopedia that contains directions in full how we may grapple with the fell destroyer, and invariably overcome him. The new rules are inflexible, the results are certain, there can be no such thing as failure. This volume is placed in our hands; we devour it greedily, and find it sweet to the tongue, but bitter to the belly: for alas, failures are as frequent as ever, unless perchance the authors of these modern ideas do the work.

We now have the Plastic or "New Departure" theory; the Cohesive and Contour Filling; the Pivot Tooth; the Electric Mallet; the new Alloy Base Plate; the Gold-lined Vulcanite Plate; and many other inventions; all and each being spread out before us as the one thing needful. Amidst this rush for new things, the writer would ask you to pause a moment, and remember that "all is not gold that glitters." He realizes that, with a large and varied experience, he has found no high road to knowledge, and no infallible rule in any branch of our art; he therefore feels justified in selecting for his theme:

MY OWN REPEATED FAILURES.

Perhaps a portion of these failures may be familiar, here and there, to some of you. If so, the writer may discover some sympathising friend or friends, into whose ear he can whisper his troubles and trials—men of like infirmities. We would not remain alone—alone in the solitude of the forest or wilderness—alone on the highway—alone in the midst of the teeming multitude—alone on the battle-field—alone between two contending forces, liable to be ground to powder between the upper and nether millstones—alone in weakness--alone in failures. May the powers forbid, and in charity remove the veil from our eyes. Mayhap we will see that they that be with us are more than they that be against us.

The dental literature of the present day is largely composed of laudation of pet theories, so much so that a disinterested stranger would be impressed with the idea that the upper rounds of the ladder of fame were well crowded, and the aspirant for honors would have to be content on the lower rounds, if he wanted room; he would come to the conclusion that there is no longer room at the top. But to encourage the weak, to whisper a word of comfort to the faint hearted, and to support the wavering, we bid you look up and take hope, for the race is not always to the swift, nor the battle to the strong. There is yet room, and plenty of room at the top. Perfection should be our aim, but we should beware how we lay claim to having attained it, lest some bright ray of truth from the great source of knowledge should reveal to all around a mass of ignorance, vanity, and error.

Turning from these preposterous claims we will now devote a short time to things of a personal character.

### MY OWN FAILURES.

Beginning our career as a dentist during the reign of Soft Foil, and in the midst of the celebrated Townsend controversy on amalgam, with not an educated dentist anywhere in reach, the duties of a practitioner were assumed. We soon found that we had failed in laying in a Technical education; that we had not learned either theory or practice, nor could we apply them the one to the other—first and most important failure, in this day inexcusable. An office is opened, and the public invited to bring their ailments for treatment. Now all the ills that flesh is heir to—-through the dental organs—toothache, abscess, periostitis, neuralgia, salivation, syphilis, dyspepsia, earache, deafness, etc., come trooping in solid phalanx, asking aid from our small stock. Some are treated with simple remedies, others dismissed as trivial and transient ailments, while the remainder are turned over to the medical practitioner as belonging to his department. As to remedies, let us see how they work. A case of toothache presents itself; something is applied, the pain is stopped, and the patient dismissed without a word-–the present trouble is bridged over. Another case on hand; extraction is requested. Without a careful examination as to the requirements of the case, at it we go-–usually with success, but often failing; the crown crushes, leaving the roots, or it may be a portion of one or more, deep seated in the alveola. Trusting to the expulsive powers of nature, we conclude to let them remain; some times they give no annoyance, while at other times they cause great suffering. Where relief is not afforded by these efforts, I record failure again.

Now, if it were possible to restore these teeth to a normal condition, and the patient were both able and willing to have it done or attempted, I have more signally failed to do my duty. Why? Because the first, therefore the most important, duty confided us is the "conservation of the teeth." 'Tis true that in many instances extraction may be necessary; but when it is not called for by plain and unmistakable terms, I may properly blame myself for doing it, and feel again that I have made a failure.

Now let us see how the matter stands with another case that presents itself. The patient comes in hurriedly, suffering greatly, and asks to have a tooth extracted. A hurried examination, reveals extensive decay; no question is asked, no suggestion is made, but the offender is removed. In a day or two the patient returns, and another tooth is taken out; but the third time he comes back designating another. Perhaps by this time a faint idea is growing in the mind of the sufferer that he will sadly miss so many teeth, and asks that an effort be made to save this one. It is done, with seeming good results. Now a disagreeable suspicion floats through my mind, that the latter tooth has been really the offender all the while. 'Tis true the others were in a bad condition, but were probably in better condition to treat successfully than the latter. If this be so, I must record failure again—knowing my duty, I did it not. Perhaps I console myself with the idea that I only did what I was asked to do. So far true; but responsibility requires that every other remedy shall be exhausted first. The patient should have been advised as to the importance of the organ for mastication, etc., and the probabilities of restoring its usefulness, the time necessary to treat it properly, and the probable expense. These things should be plainly set before the sufferer, and, with his consent, the effort honestly made. By so doing, I avoid reproach for failure. We have now before us a trial of judgment and skill in an effort to restore the tooth to a healthy condition. What shall the treatment be? The choice lies between extirpating the nerve, in part or in whole, or by treatment restore it to health, and judiciously fill. Perhaps I decide to destroy the nerve; arsenic is applied, the nerve removed, the canals enlarged, and an effort made to fill from apex to crown. In one the filling does not reach the apex, leaving an opening for the retention of fluids which eventually decompose; in another, a portion of the filling is forced through, and remains there to irritate the surrounding parts. Some times nature tolerates these conditions wonderfully, and no trouble ensues; but often trouble does ensue. The tooth is treated for a time, but has finally to be removed. Growing out of the numerous failures following this treatment, and a desire to find something better, close attention is given to treatment that may preserve the nerves in health. These efforts are confronted with many difficulties—the greatest, perhaps, being the want of knowledge as to the exact condition of the nerve, either before, during, or after treatment; but I try the plan—subdue the pain, release pus, and stimulate the nerve to throw off disease—every thing seems favorable. I then fill, but to find out sooner or later that either I have made a mistake as to condition, or have committed a blunder in mode or manner of filling. So, in this line I find failures of frequent occurrence. I am somewhat reconciled to this experience when I remember that I can, as a last resort, try the first plan, thus giving the tooth two chances.

Now we have not related all the failures that ensue from either plan, although a large majority of such operations may be classed as a success, as they are not heard from again. Yet even among those classed as successful, I am satisfied if I knew their history I would be less confident of their success. Then in plain and compound fillings, here and there, now and then, with napkin, spunk, bibulous paper, or rubber dam; with gold, soft or cohesive, singly or combined; with amalgam, gutta percha, or any of the cements; with wedge-shaped or serrated pluggers, hand-pressure or mallet, a filling fails through some fault of mine--I can not hide it from myself—yet how is it that I am alone in this experience?

It falls to my lot to have a child put under my care at an early age; the teeth are soft, I clean and fill, give careful directions as to how best to preserve them, and the directions are carried out; every six months I examine, fill, file, and clean; but alas, one and another of the teeth give away, and finally, perhaps long ere middle age has been reached, every tooth is out and their places supplied with shop teeth; while with a feeling of relief the martyr to dentistry utters a thank the Lord, they are all gone, and goes out to extol the merits of the substitute. Here, too, I appropriately inscribe failure on my banner.

I am told that my failures in filling arise from not preparing my cavities well; that I cut away too much or too little: that I do or do not use retaining points; that I put in too much or too little gold; that I should use soft foil; that I should use cohesive; that I should use amalgam, or some other plastic; that I never should allow myself to use amalgam, etc. Bewildered, I drop my wedge-shaped pluggers and soft foil, I turn to serrated instruments; work them by hand, then by the automatic, followed by the hand-mallet, from two ounces to a pound in weight; then the electric plugger. I give large V shaped separations--on Arthur's style--then I wedge or drill from crown, in hopes of hitting the line of an approximal cavity--may miss it some, but can finally succeed by enlarging enough. What does the loss of so much structure amount to? Nothing, when the tooth is saved; but as I sometimes lose one of these fillings, I often find at second trial that the tooth is gone, sacrified to the theory of no separations. The result, of course, is due to imperfect work.

I discard amalgam, but to take it up again; change my mode of work, but to either try something new, or go back to some old and well-tried method, and be content to learn that each and every plan that has yet been devised to save the teeth will be accompanied with numerous failures, if I have any thing to do with them.

I might tell you how I have labored with abscessed teeth and the varying results--sometimes curing the abscess, restoring the teeth to usefulness for a while, but the trouble finally returning; removing necrosed or denuded alveolæ; excising the denuded portions of roots; of efforts to adapt artificial dentures to the mouth--and the many marked instances of failure; pivoting by the old method, by Peabody's, Richmond's, Bonwill's and others, and the failures that follow; riveting or banding split teeth; controlling toothache, neuralgia, correctly diagnosing odontoblasts, and other calcific matter in pulp cavities, pulpitis, periostitis, or exostosis, pyorrhœa alveolaris, or senile decay--in fact, in every department of the profession I have, and do make repeated failures, both in judgment and skill, and so confess to you, and express my humiliation at the fact and an earnest desire to avoid them. It is true that sometimes I catch a glimpse of what looks like a failure, coming from those who are eminent and have impressed me with their infallibility. But this very confession will bar my evidence, as I have shown you how liable I am to err; so that you may readily believe that I am unable to distinguish between success and failure.

Now, sirs, while I have made these confessions, I have avoided any allusion to the other side of the subject. I appeal to you all to observe well, and see if we are justified in taking as strong positions as we usually do in defense of our peculiar modes. Will the facts sustain us? If not, let us retrace our steps and take a position that we can maintain, lament our short comings and try to overcome them, be not too confident or boastful lest we come to grief, and ever bear in mind that in each and every plan there is more or less that is good and should be retained for use at the proper time and place. Do not let us limit our resources, but be prepared to draw forth things new and old, and apply them to the credit of the profession and the good of humanity.

